# Cross-Dataset Facial Micro-Expression Recognition with Regularization Learning and Action Unit-Guided Data Augmentation

**DOI:** 10.3390/e28020150

**Published:** 2026-01-29

**Authors:** Ju Zhou, Xinyu Liu, Lin Wang, Tao Wang, Haolin Xia

**Affiliations:** 1 Shenzhen Institutes of Advanced Technology, Chinese Academy of Sciences, Shenzhen 518055, China; zhouju@szpu.edu.cn (J.Z.); lin.wang1@siat.ac.cn (L.W.); 2 Tech X Academy, Shenzhen Polytechnic University, Shenzhen 518055, China; 3 School of Computer Science and Artificial Intelligence, Guangdong University of Education, Guangzhou 510303, China; lxinyu08@gdei.edu.cn; 4 Shenzhen Lower Limb Intelligent Rehabilitation Engineering Research Center, Shenzhen 518055, China; 5 Hong Kong Center for Construction Robotics, Hong Kong 999077, China; xiahl@ust.hk

**Keywords:** micro-expression recognition, cross-dataset recognition, regularization learning, action unit, data augmentation

## Abstract

With the growing development of facial micro-expression recognition technology, its practical application value has attracted increasing attention. In real-world scenarios, facial micro-expression recognition typically involves cross-dataset evaluation, where training and testing samples come from different datasets. Specifically, cross-dataset micro-expression recognition employs multi-dataset composite training and unseen single-dataset testing. This setup introduces two major challenges: inconsistent feature distributions across training sets and data imbalance. To address the distribution discrepancy of the same category across different training datasets, we propose a plug-and-play batch regularization learning module that constrains weight discrepancies across datasets through information-theoretic regularization, facilitating the learning of domain-invariant representations while preventing overfitting to specific source domains. To mitigate the data imbalance issue, we propose an Action Unit (AU)-guided generative adversarial network (GAN) for synthesizing micro-expression samples. This approach uses K-means clustering to obtain cluster centers of AU intensities for each category, which are then used to guide the GAN in generating balanced micro-expression samples. To validate the effectiveness of the proposed methods, extensive experiments are conducted on CNN, ResNet, and PoolFormer architectures. The results demonstrate that our approach achieves superior performance in cross-dataset recognition compared to state-of-the-art methods.

## 1. Introduction

Micro-expressions refer to involuntary facial movements that reveal concealed emotions, characterized by their brief duration and low intensity. Due to their spontaneous nature, micro-expressions serve as a critical cue for inferring true emotions [1,2,3]. Since their introduction, they have been extensively studied across disciplines including psychology and sociology. However, accurately detecting micro-expressions with the naked eye is challenging for non-experts, which has motivated the development of computer-based automated micro-expression analysis [4,5,6]. As a key technology in human affective computing, micro-expression recognition holds significant promise in critical fields such as suicide prevention [7], criminal interrogation [8], and national security [9].

The advancement of micro-expression recognition research relies fundamentally on the support of well-annotated datasets. Despite the considerable challenges given the spontaneous and subtle nature of micro-expressions, persistent research efforts have yielded several valuable open-source datasets, including SMIC [10], CASME [11], CASME II [12], SAMM [13], and MMEW [14]. These datasets provide an essential foundation for model training and evaluation. However, existing datasets still face critical limitations: the total number of publicly available samples is relatively small (less than 2000 in total), which restricts model complexity and generalizability, and significant class imbalance across emotion categories further complicates the development of robust recognition. These issues pose challenges to building reliable micro-expression recognition models.

Although many effective micro-expression recognition models have been developed using existing datasets, the prevailing methodology remains largely dependent on an intra-dataset evaluation protocol, wherein models are trained and tested on data from the same source [5,8]. This approach ensures highly consistent feature distributions between training and testing splits, which facilitates learning but also causes models to overfit to dataset-specific statistical biases. In real-world applications, however, models are required to generalize to data collected under different conditions with divergent quality and feature distributions. Under such circumstances, conventional models developed under the intra-dataset paradigm typically suffer a significant performance degradation, revealing a critical lack of generalization capability [15].

**Figure 1:**
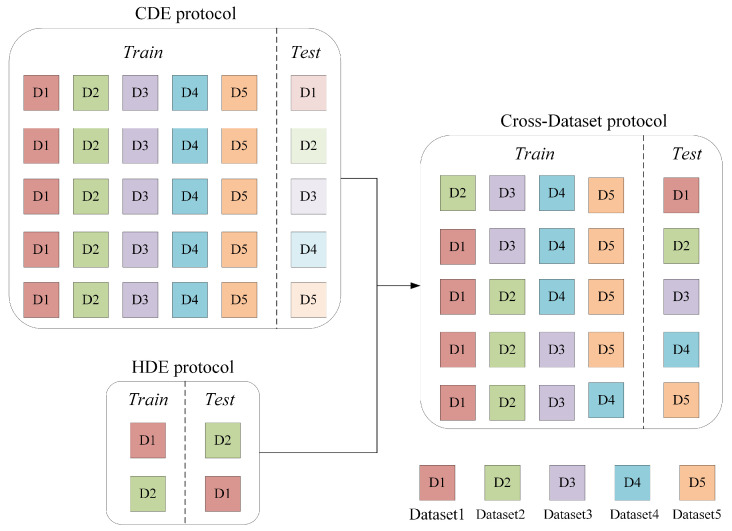
Schematic illustration of different micro-expression recognition evaluation protocols.

To address the generalization challenge in micro-expression recognition, the Micro-Expression Grand Challenge 2018 (MEGC2018) and 2019 (MEGC2019) introduced two protocols: Composite Dataset Evaluation (CDE) [16] that merges multiple datasets for training and testing to learn general features, and Holdout Dataset Evaluation (HDE) [17] that trains on one dataset and tests on another to simulate cross-dataset scenarios. For CDE, Liu et al. integrate optical flow motion analysis, structural feature pooling, and cross-domain knowledge transfer to align feature distributions across source datasets [18]. For HDE, Zong et al. propose a Target Sample Re-Generator on the CASME II and SMIC datasets. This method generates target samples with a feature distribution similar to the source domain to minimize distribution discrepancies [19]. Although these methods have achieved promising results, as shown in the left part of Figure 1, existing evaluation protocols suffer from notable limitations in assessing model generalizability. The CDE protocol, despite its ability to learn distribution discrepancies across multiple source datasets, does not evaluate models on truly unseen data. Meanwhile, conventional HDE setups typically utilize only two datasets, which prevents models from being exposed to sufficiently diverse sample distributions. Consequently, the generalizability of models trained under these paradigms is often limited [20].

To overcome these limitations and better simulate real-world conditions, we propose a comprehensive cross-dataset micro-expression recognition framework based on a leave-one-dataset-out strategy across five datasets. For instance, Datasets 2–5 are combined for training, while Dataset 1 is held out for testing. As shown in the right part of Figure 1, this approach maximizes data utilization while ensuring rigorous evaluation on completely unseen data distributions, thereby closely mimicking real-world scenarios where models are required to generalize to data from entirely new sources.

While this framework significantly enhances evaluation rigor, it simultaneously amplifies inherent challenges in cross-dataset recognition. The first challenge stems from feature distribution inconsistency. Disparities in acquisition hardware, illumination environments, and subject populations create substantial distribution shifts across datasets. When multiple datasets are merged for training, these discrepancies not only intensify internal feature variations within the training set but also magnify the distribution gap with the held-out test set. The second challenge is dataset imbalance. Each dataset exhibits unevenness in both quantity and class distribution, and combining multiple datasets further intensifies this imbalance. This composite imbalance may lead the model to over-rely on datasets or classes with larger sample sizes during training, thereby reducing its generalization capability across different data sources. We provide a detailed visual analysis of these cross-dataset challenges in Section 2.

To address feature distribution inconsistency in cross-dataset recognition, we propose a distribution-balanced batch regularization learning (BRL) approach. Implemented as a specialized loss component through a self-attention mechanism, this method establishes dataset grouping constraints in the feature space and minimizes inter-group attention weight differences to force balanced feature learning from all source domains. The BRL module acts as an information flow regularizer, minimizing the entropy disparity in cross-domain attention distribution and encouraging the extraction of domain-invariant features with higher mutual information to emotion labels. Experiments demonstrate that BRL module effectively prevents overfitting to individual datasets and significantly improves cross-dataset generalization. To address data imbalance, we propose a data augmentation method based on Action Unit (AU) intensity clustering. By analyzing the AU intensity distribution of the same emotion category across different datasets, we extract representative cluster centroids as average AU weights to guide the ULME-GAN network [21] in generating semantically consistent samples that align with the real data distribution. By effectively expanding samples, this strategy enhances the information diversity of the training set while preserving the essential statistical properties and semantic plausibility of micro-expressions. Comprehensive experiments using three mainstream networks (CNN, ResNet, and PoolFormer) on five spontaneous datasets (SMIC, CASME, CASME II, SAMM, and MMEW) demonstrate substantial performance improvements of our approach over state-of-the-art methods under the rigorous cross-dataset evaluation protocol.

The main contributions of this study are summarized as follows:

(1) A rigorous cross-dataset evaluation protocol is established for micro-expression recognition, systematically addressing key challenges through targeted solutions while achieving state-of-the-art performance under this realistic setting.

(2) A batch regularization learning strategy is proposed to address feature distribution inconsistency across datasets, implemented as a loss term that explicitly balances the model’s attention to different datasets at the representation learning level.

(3) An AU-guided data generation strategy is developed, which not only effectively mitigates class imbalance but also maintains the authenticity of data distributions, contributing to improved model performance.

The rest of this paper is organized as follows: Section 2 reviews related work. Section 3 provides a systematic analysis of cross-dataset challenges through visualizations and elaborates the proposed targeted solutions. Section 4 presents the experimental results and discussions. Section 5 concludes this study and suggests future research directions.

## 2. Related Work

With the advancement of micro-expression recognition research, particularly since the launch of the Micro-Expression Grand Challenge, the research focus has progressively shifted from single-dataset evaluation to CDE and HDE validation paradigms that emphasize generalization capability [22,23,24,25].

Under the CDE paradigm, the main goal is to learn highly generalizable feature representations from the mixed distribution of multiple data sources. Research primarily follows three technical paths: transfer learning, domain adaptation through feature distribution alignment, and data augmentation to address scarcity. For example, representative work includes a three-stage transfer learning framework proposed by Peng et al., which fine-tunes a network first on ImageNet, then on macro-expression datasets, and finally on micro-expression datasets, significantly improving model adaptation to composite data [26]. Furthermore, Yu et al. develop ICE-GAN, which uses a generative adversarial network to synthesize micro-expression samples with controllable attributes, offering a new approach to mitigate data scarcity [27]. Zhang et al. propose a Global–Local Feature Fusion Network (GLFNet) that integrates global attention and local block modules with an adaptive feature fusion mechanism, and employs a class-balanced loss to effectively address the challenges of subtle motion and class imbalance in micro-expression recognition [28]. Gan et al. propose a network called MAG, which aligns macro-expressions with micro-expressions based on action similarity [29]. By integrating nonlinear amplification and guidance mechanisms to enhance feature saliency, MAG improves the recognition performance of CDE. Zhang et al. propose a Hierarchical Feature Aggregation Network (HFA-Net), which further enhances micro-expression recognition performance through multi-level feature aggregation and adaptive attention feature fusion [30].

Under the HDE paradigm, the research focus is on effective knowledge transfer from known source domains to completely unseen target domains. In addition to domain-adaptive distribution alignment methods, research in this area also encompasses targeted feature design and selection, as well as enhancement techniques for local discriminative features. For example, Peng et al. propose the Apex-Time Network (ATNet), a novel framework that leverages spatial information from the apex frame and temporal cues from adjacent frames, systematically validating the effectiveness of spatiotemporal fusion and demonstrating the critical role of temporal features in improving model generalization [31]. Mao et al. propose a Region-Relational Reasoning Network (RRRN) for occluded micro-expression recognition. This network enhances model robustness by modeling inter-region relationships and employing an attention mechanism to mitigate occlusion effects [32].

Recently, fully cross-dataset micro-expression recognition has emerged to better simulate real-world application scenarios. This paradigm requires models trained on multiple source domains to perform well directly on completely unseen target datasets. Researchers explore various advanced technical routes to address this challenge. These include stability feature design based on facial regions of interest, data augmentation, meta-learning for rapid domain adaptation, and many innovative network architectures. For example, Zhang et al. develop the Region-Selective Transfer Regression (RSTR) method to significantly improve cross-dataset recognition performance by concentrating on facial local regions that exhibit high cross-dataset consistency [33]. To mitigate the feature distribution discrepancy across databases, Zong et al. develop a domain regeneration approach capable of synthesizing new micro-expression samples, thereby narrowing the domain shift between source and target datasets [34]. Addressing the issue of intra-class variation in micro-expressions, Wang et al. introduce MCNet, a meta-clustering learning network designed to enhance recognition performance [35].

However, existing studies show that current cross-dataset methods predominantly adopt general strategies from CDE and HDE paradigms, lacking specialized optimization for the distinctive requirements of cross-dataset scenarios. In particular, the inherent feature distribution discrepancies in multi-source domain training and the intrinsic data imbalance in composite training sets still lack systematic visualization analysis and targeted solutions. The prevailing reliance on either transferring generic approaches or constructing overly sophisticated models fails to address these fundamental issues, compromising practical stability and scalability in real-world applications. Therefore, by clearly identifying the key challenges in cross-dataset micro-expression recognition, this paper proposes a batch group regularization constraint and an action unit-guided data balancing method to provide more targeted technical solutions.

## 3. Analysis of Cross-Dataset Challenges and Proposed Methods

As illustrated in Figure 1, this study investigates a more challenging cross-dataset recognition paradigm that integrates characteristics of both CDE and HDE protocols. We begin by conducting comprehensive analysis to identify and characterize two critical challenges in this rigorous evaluation protocol: feature distribution inconsistency across source domains and inherent data imbalance. Building on these analysis, we subsequently present a targeted solution framework incorporating Batch Group Regularization for domain-invariant representation learning and AU-guided data augmentation for dataset rebalancing.

### 3.1. Analysis of Cross-Dataset Challenges

The adoption of CDE and HDE evaluation protocols has improved model generalization to some extent. However, in the context of rigorous cross-dataset recognition involving large-scale multi-dataset scenarios that better reflect real-world conditions, this paradigm amplifies two inherent challenges: feature distribution inconsistency and data imbalance.

#### 3.1.1. Feature Distribution Inconsistency

Feature distribution differences are a core challenge in cross-dataset micro-expression recognition. These differences come from variations in how datasets are collected, including the capture devices, experimental conditions, and participant groups. From an information-theoretic perspective, these variations introduce domain-specific biases that increase the uncertainty (or entropy) of the composite feature space, making it difficult for models to extract stable, discriminative signals for emotion recognition.

(1) Data Acquisition Differences

As summarized in Table 1, the five benchmark datasets employed in this study exhibit significant differences in acquisition specifications. Camera frame rates range from 60 fps to 200 fps, while facial region resolutions vary from 150 × 190 to 400 × 400 pixels. These technical discrepancies directly introduce heterogeneity in both spatiotemporal information density and image detail across datasets. This heterogeneity can be viewed as differing informational content per sample across domains. For instance, a higher frame rate (e.g., 200 fps) provides a denser temporal signal, potentially carrying more information about the dynamics of a micro-expression, whereas variations in resolution affect the spatial information entropy of the facial details.

**Table 1:** Comparison of the five spontaneous micro-expression datasets used in this study.

Datasets	Frame Rate (fps)	Facial Resolution	Sample Size Per Category			Total
			**Positive**	**Negative**	**Surprise**	
SMIC [10]	100	190 × 230	51	70	43	164
CASME [11]	60	150 × 190	9	53	21	83
CASME II [12]	200	280 × 340	32	72	25	129
SAMM [13]	200	400 × 400	26	80	15	121
MMEW [14]	90	400 × 400	36	109	89	234

(2) Experimental Environment and Ethnicity

**Figure 2:**
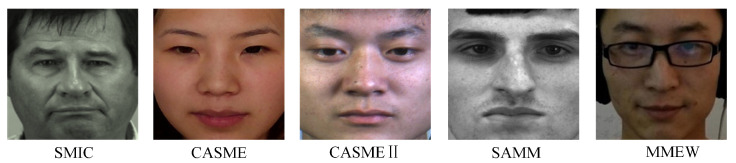
Sample images of the “happiness” category from different datasets.

Besides equipment parameters, differences in experimental environments introduce systematic biases in image characteristics across datasets. These environmental factors include lighting conditions, experimental settings, and participant ethnicity. As shown in Figure 2, “happiness” micro-expression samples from five different datasets exhibit noticeable differences in illumination characteristics, lighting intensity, and skin tone. These visual variations are unrelated to micro-expression muscle movements, yet deep neural networks may learn them as shortcut features during training. Such shortcut learning interferes with the acquisition of generalizable micro-expression features for cross-dataset transfer, ultimately limiting performance in practical applications.

(3) Evidence from Feature Visualization

**Figure 3:**
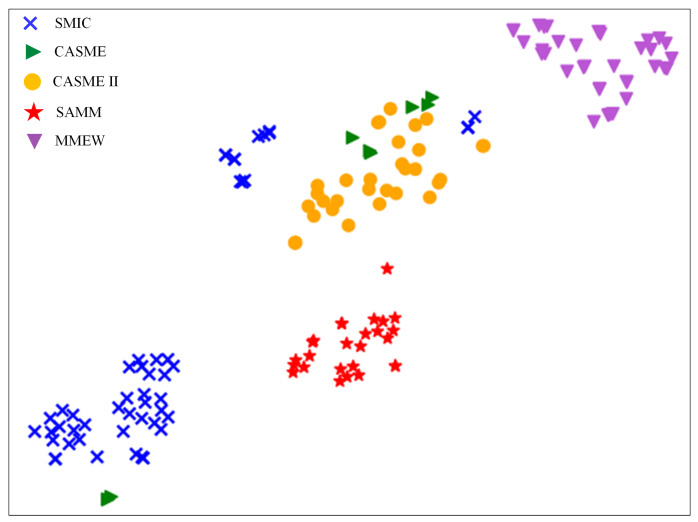
Visualization of the feature space distribution for “happiness” samples from different datasets.

To evaluate feature distribution inconsistency in cross-dataset scenarios, we train a convolutional neural network using all “happiness” category samples from these five datasets and visualize their distributions in the feature space. Specifically, we project the high-dimensional feature representations into a two-dimensional space for visualization using t-SNE [36]. As shown in Figure 3, the results clearly demonstrate that samples from the same dataset form tight clusters, while clear distribution gaps exist between different datasets. This finding indicates that as more datasets are combined for training, the feature distribution differences across source domains become more pronounced. In this cross-dataset paradigm, models struggle to learn unified semantic representations, and the intensified distribution inconsistency leads to isolated feature subspaces, thereby limiting generalization performance on unseen datasets.

The visualization in Figure 3 also reveals a critical information structure problem. The strong intra-dataset clustering and inter-dataset separation imply that the learned features are highly predictive of their source dataset domain, sharing substantial mutual information with the dataset identity, but are less informative about the micro-expression emotion category. Ideally, for robust cross-dataset recognition, the features should be highly informative of the emotion label while being invariant to the dataset source. This misalignment increases the uncertainty in predicting the emotion from the features, making accurate classification across domains challenging. Therefore, our proposed method aims to rectify this by guiding the model to learn features that are more discriminative for emotions and less dependent on the dataset origin.

#### 3.1.2. Data Imbalance

Beyond feature distribution differences, the imbalance in data size across datasets presents another important challenge. This issue mainly appears in two aspects: overall data quantity differences and uneven distribution at the category level.

(1) Overall Data Quantity Differences

As shown in Table 1, the five datasets comprise a total of 731 samples, but their distribution is highly uneven. For example, the MMEW dataset contains 234 samples, while CASME has only 83 samples. This quantity difference causes models to over-rely on larger datasets during composite training, leading to overfitting. Consequently, the model performs well on certain datasets but degrades significantly on others. This can be interpreted as the model allocating disproportionate attention entropy or computational resources to domains with higher sample counts, essentially forming an inefficient information acquisition strategy that fails to maximize knowledge from all available sources.

(2) Class Distribution Imbalance

A more serious problem is the imbalance at the class level. The number of samples from different datasets varies greatly across emotion categories. For instance, in the “Positive” category, SMIC has 51 samples while CASME has only 9 samples; in the “Surprise” category, MMEW has 89 samples while SAMM has only 15 samples. This class imbalance results in certain categories being predominantly represented by limited datasets during training. Consequently, learned features reflect characteristics of these dominant datasets rather than essential micro-expression attributes, which significantly undermines the model generalization across datasets.

### 3.2. Proposed Methods

**Figure 4:**
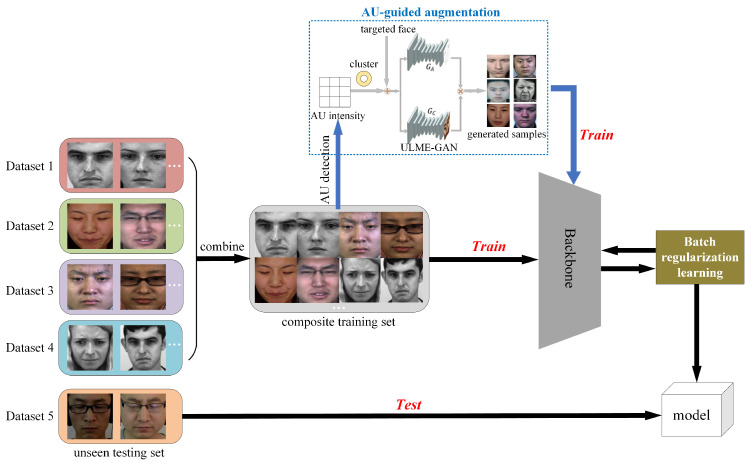
The overall architecture of the proposed method.

Figure 4 presents the overall architecture of the proposed cross-dataset micro-expression recognition framework. The method employs five spontaneous micro-expression datasets in a leave-one-dataset-out protocol: four datasets form a composite training set, while the remaining unseen dataset serves as the test set to evaluate model generalization. Taking input apex frames as an example, the framework first performs AU detection and clustering analysis to construct category-specific AU intensity matrices. These matrices subsequently guide the ULME-GAN [21] in generating augmented micro-expression samples, as illustrated in the blue dashed box of Figure 4 and detailed in Section 3.2.2. Both original and augmented samples are processed through a backbone network for feature extraction. During training, a batch regularization learning module optimizes the process by computing a regularization loss via self-attention mechanism. This BRL loss is combined with cross-entropy loss through weighted summation to form the final optimization objective. The trained model is ultimately evaluated on the completely unseen test dataset to assess cross-dataset recognition performance.

#### 3.2.1. Batch Regularization Learning (BRL)

In cross-dataset micro-expression recognition, models trained on composite datasets often develop biased feature representations due to distribution discrepancies among source domains. This bias manifests as overfitting to certain datasets while underfitting others, ultimately compromising generalization performance. From an information-theoretic perspective, this bias represents an inefficient allocation of the model’s information acquisition capacity—it extracts excessive information from some domains while neglecting others, leading to suboptimal information integration across sources. To address this challenge, we propose the BRL module that explicitly balances the model’s attention distribution across different datasets during training, ensuring a more equitable information flow from all source domains.

**Figure 5:**
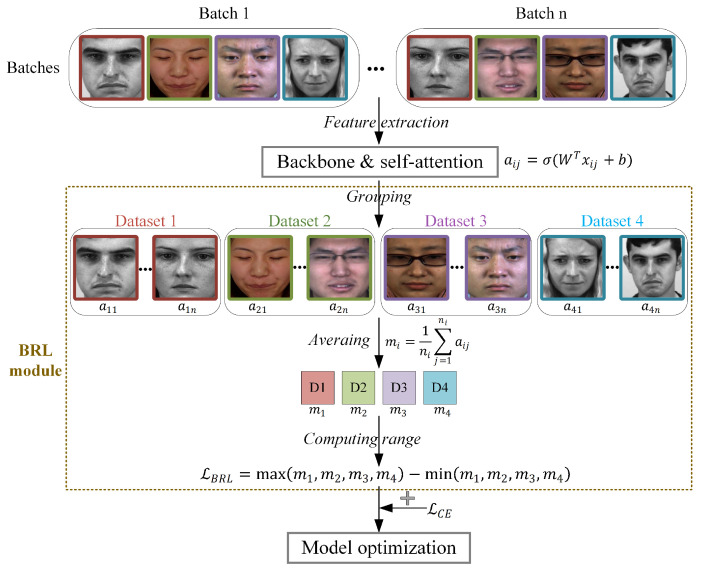
The computational workflow of the BRL module.

The BRL module consists of two key components: a self-attention weighting mechanism and group-wise regularization learning. Figure 5 illustrates the computational workflow of the proposed BRL module.

First, the self-attention mechanism [37] computes sample-wise importance weights based on the extracted features. Let $x_{ij}\in R^{D}$ denote the feature vector of the *j*-th sample from the *i*-th dataset, where *D* is the feature dimension. This feature vector is projected through a fully connesxcted layer to obtain a self-attention weight:

(1) $$a_{ij}=\sigma \left(W^{⊤}x_{ij}+b\right)$$

where $a_{ij}$ is the computed self-attention weight, *W* and *b* denote the weight matrix and bias term of the fully connected layer, and σ is the sigmoid activation function. The magnitude of $a_{ij}$ indicates the sample’s relative contribution to model learning, where higher values correspond to greater influence. Conceptually, $a_{ij}$ quantifies the information gain the model attributes to each sample during the learning process.

The regularization component utilizes these attention weights to promote balanced learning across different source datasets. During training, samples in each batch are grouped according to their source dataset, and the average attention weight $m_{i}$ is computed for each group as follows:

(2) $$m_{i}=\frac{1}{n_{i}}\sum_{j=1}^{n_{i}}a_{ij},\;i\in 1,2,3,4$$

where $n_{i}$ denotes the number of samples from the *i*-th dataset in the current batch, and $m_{i}$ represents the mean attention weight for that dataset. Here, $m_{i}$ serves as a proxy for the average information contribution from domain *i* to the current learning step. A balanced set of $m_{i}$ values indicates that the model is extracting knowledge uniformly from all available datasets, thereby encouraging the learning of domain-invariant representations. Conversely, the imbalanced set of $m_{i}$ values suggest that the model’s learned features are likely biased toward specific domains, containing dataset-specific information that hinders cross-dataset generalization.

The BRL module functions through a loss term $L_{BRL}$, which is defined as the range between the maximum and minimum of these dataset-wise average attention weights:

(3) $$L_{BRL}=H-L$$

where $H=\max(m_{1},m_{2},m_{3},m_{4})$ and $L=\min(m_{1},m_{2},m_{3},m_{4})$ represent the highest and lowest average attention weights among the four dataset groups, respectively. By minimizing this range-based loss, the module explicitly penalizes attention bias to any particular dataset, thereby enforcing more balanced feature learning across all source domains and encouraging the discovery of domain-invariant representations. Minimizing H−L is equivalent to reducing the entropy (or variance) in the distribution of information contributions $\left\{m_{i}\right\}$ across domains. This regularization encourages the model to become a fair information processor that extracts knowledge uniformly from all sources. In cross-dataset micro-expression recognition, balanced attention compels the model to focus on shared emotion-related semantics across datasets, thereby avoiding overfitting to non-transferable, dataset-specific artifacts such as illumination, resolution, or demographic variations.

The overall optimization objective integrates the proposed BRL loss $L_{BRL}$ with the standard multi-class cross-entropy loss $L_{CE}$ to jointly optimize classification performance and cross-dataset generalization. The complete loss function $L_{total}$ combines both objectives through setting weights:

(4) $$L_{total}=\left(1-\lambda \right)L_{CE}+\lambda L_{BRL}$$

where λ∈[0,1) is a balancing coefficient that controls the relative contribution of each loss component. This formulation ensures that the cross-entropy loss $L_{CE}$ always maintains a non-zero weight to preserve classification capability, while the batch regularization loss $L_{BRL}$ provides adjustable constraint strength for feature distribution alignment. This dual-objective framework can be interpreted as simultaneously minimizing the prediction uncertainty (via cross-entropy) and regularizing the information acquisition strategy (via BRL) to promote domain invariance.

Through the optimization of this composite objective, the model simultaneously maintains discriminative power for accurate classification while learning to allocate attention resources equitably across all source domains. This dual optimization strategy promotes the learning of domain-invariant feature representations that capture essential micro-expression characteristics while minimizing dataset-specific biases, ultimately enhancing generalization capability in cross-dataset scenarios. The resulting features exhibit higher mutual information with the true emotion labels and lower dependence on dataset identifiers, aligning with the information-theoretic principle of learning maximally informative yet minimally domain-specific representations.

Designed as a plug-and-play component, the BRL module operates at the mini-batch level, enabling application to diverse cross-domain learning tasks beyond micro-expression recognition. Its batch-wise processing mechanism allows seamless integration into standard deep learning pipelines without requiring modifications to the host network architecture. This modular design establishes a portable framework for enhancing model robustness across various cross-dataset applications. More broadly, it offers a principled, information-theoretic approach to mitigating source bias in any multi-source learning scenario by explicitly regularizing the information flow during training.

#### 3.2.2. AU-Guided Micro-Expression Augmentation

As shown in Table 1, the five spontaneous micro-expression datasets employed in this study exhibit significant data imbalance, which can bias the model to majority classes and impair its generalization capability. To address this issue while ensuring the authenticity of synthesized samples, we propose an AU-guided data augmentation strategy that generates semantically consistent micro-expression samples aligned with the real data distribution.

The proposed method builds upon the ULME-GAN framework [21], which leverages a combination of adversarial loss, attention loss, conditional expression loss, and identity loss to ensure the generation of realistic and identity-consistent micro-expression sequences. Our approach extends this framework by incorporating AU intensity clustering to guide the generation process.

Specifically, for each emotion category in the composite training set, we first extract AU intensity values from all available samples using OpenFace [38]. The intensity vector for the *p*-th sample is defined as:

(5) $$\mathrm{AU}p=\left[I_{\mathrm{AU}1}^{\left(p\right)},I_{\mathrm{AU}2}^{\left(p\right)},\ldots ,I_{\mathrm{AU}45}^{\left(p\right)}\right],\;p\in \left[1,M\right]$$

where $I_{\mathrm{AU}k}^{\left(p\right)}$ denotes the intensity value of the AU*k* for sample *p*, with *k* representing the specific AU identifier (1, 2, 4, 5, 6, 7, 9, 10, 12, 14, 15, 17, 20, 23, 25, 26, and 45), and *M* represents the total number of samples in the current emotion category. The complete AU intensity set for the category is then:

(6) $$A=\{{\mathrm{AU}}_{1},{\mathrm{AU}}_{2},\ldots ,{\mathrm{AU}}_{M}\}$$

To obtain a representative AU pattern for each emotion category, we apply K-means clustering to the AU intensity set A and identify the optimal cluster centroid. The centroid vector ${\mathrm{AU}}_{c}$ is computed as the mean of all AU intensity vectors in the dominant cluster, representing the prototypical AU activation pattern for that emotion category. This centroid serves as the conditional input to ULME-GAN, guiding the generation of synthetic samples that embody the characteristic AU configuration of the category while maintaining semantic consistency with real micro-expressions.

The generation process follows the adversarial training framework, where the generator *G* learns to transform neutral face images into expression-carrying faces conditioned on the centroid vector ${\mathrm{AU}}_{c}$. The training objective incorporates multiple loss components: adversarial loss ($L_{\mathrm{adv}}$) to ensure visual realism, attention loss ($L_{\mathrm{att}}$) to preserve spatial coherence, conditional expression loss ($L_{\exp}$) to maintain expression fidelity, and identity preservation loss ($L_{\mathrm{id}}$) to retain subject characteristics.

To establish a balanced dataset across all emotion categories, we use the MMEW dataset as the reference due to its largest sample size of 234 samples. Each dataset undergoes augmentation to reach this target, with generated samples distributed evenly across all emotion categories. Table 2 provides the detailed numbers of augmented samples required for each dataset. It should be noted that the MMEW dataset serves as the benchmark in this process and therefore does not require additional augmentation.

This AU-guided augmentation strategy offers significant advantages by directly utilizing the clustered AU intensity vectors as the standard for data generation. This approach ensures that the synthesized samples accurately reflect the most representative and prototypical facial muscle movement patterns for each emotion category, effectively capturing the essential characteristics of genuine micro-expressions. By employing these statistically meaningful AU patterns derived from real data distributions, our method not only effectively mitigates data imbalance across datasets but also maintains high semantic authenticity in the generated samples. Consequently, the augmentation process enhances the model’s capacity to learn robust and domain-invariant feature representations that generalize well across all source domains.

#### 3.2.3. Backbone Networks

**Figure 6:**
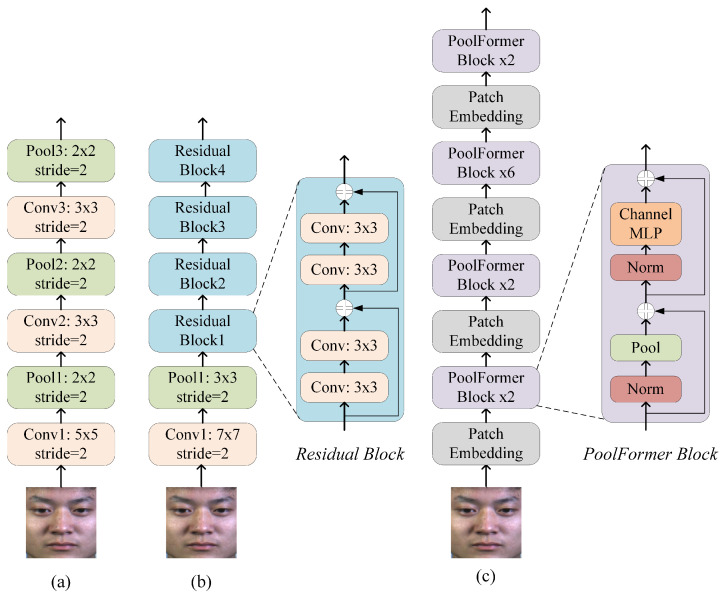
The backbone network architectures of the (**a**) CNN, (**b**) ResNet, and (**c**) PoolFormer.

To validate the effectiveness of our proposed methods independently of network architecture complexity, we employ three representative backbones: CNN, ResNet, and PoolFormer. This selection ensures that performance improvements stem from our proposed BRL and AU-guided augmentation approaches rather than sophisticated network designs. As shown in Figure 6, these widely-used backbone networks provide diverse architectural paradigms for micro-expression recognition.

(1) CNN

The CNN backbone, illustrated in Figure 6a, consists of a sequence of convolutional and pooling layers for hierarchical feature extraction. Input images are processed using convolutional kernels of sizes 5×5 and 3×3, each followed by a 2×2 pooling layer to progressively reduce spatial resolution and enhance translational invariance. The extracted features are flattened and passed through a fully connected layer to produce predictions across the three target emotion categories. This simple yet effective architecture serves as a baseline to evaluate the generalizability of our proposed methods.

(2) ResNet

The ResNet backbone is originally proposed to address the issues of vanishing gradients and network degradation in very deep convolutional networks [39]. As illustrated in Figure 6b, we employ ResNet-18 in this study, a lightweight variant of the architecture. The core residual building block utilizes skip connections that bypass one or more convolutional layers, enabling a direct, element-wise addition between the input and the transformed feature. This design establishes a residual learning framework that effectively alleviates the vanishing gradient problem in deep networks and has demonstrated strong performance across various recognition tasks.

(3) PoolFormer

PoolFormer represents a transformer-type architecture that substitutes the standard self-attention mechanism with spatial pooling operations [40]. This design significantly lowers computational cost while maintaining competitive performance. As illustrated in Figure 6c, we adopt the PoolFormer-S12 configuration in this study, which stacks 12 layers of identical PoolFormer blocks. Each block applies a 3×3 average pooling layer to mix spatial information, followed by channel-wise MLP modules and residual connections. The network progressively reduces spatial resolution while increasing channel dimensions across four stages. This efficient architecture provides a practical balance between accuracy and speed, making it suitable for micro-expression recognition tasks where computational efficiency and cross-dataset generalization are both important.

## 4. Experimental Results and Analysis

In this section, we present a comprehensive evaluation of the proposed method to validate its effectiveness. We begin by describing the experimental setup, including descriptions of the datasets employed, the data preprocessing pipeline, and the implementation details. Subsequently, the evaluation metrics used for quantitative analysis are defined. A thorough analysis of the results is then provided, encompassing an ablation study to dissect the contribution of each proposed component, followed by a comparative analysis with the state-of-the-art methods.

### 4.1. Datasets

This study employs five publicly available spontaneous micro-expression datasets (SMIC [10], CASME [11], CASME II [12], SAMM [13], and MMEW [14]) to establish a comprehensive benchmark for cross-dataset evaluation. Except for SMIC, all other datasets provide apex frame annotations and corresponding AU annotations. For SMIC, we followed established research conventions by using the frame with the largest detected motion magnitude as the apex frame. Each micro-expression dataset contains 3 to 8 emotion categories. To maintain categorical consistency across datasets with different original emotion labels, all samples are mapped into three emotion categories: Positive, Negative, and Surprise.

As illustrated in Figure 1 and detailed in Table 3, the cross-dataset evaluation follows a leave-one-dataset-out protocol. For each test round, one dataset is held out as the test set, while the remaining four are combined to form a composite training set. This process is repeated five times, ensuring each dataset serves as the test set. It is important to note that all augmented samples are used exclusively for training and do not participate in the testing phase. The original sample sizes for each test dataset are provided in Table 1.

Table 3 details the sample composition of the composite training sets corresponding to each testing scenario, comparing the original and augmented sample sizes. The data augmentation strategy effectively balances the training distribution, with each composite set reaching 936 samples after augmentation, significantly enhancing the model’s exposure to diverse sample distributions.

### 4.2. Data Preprocessing and Implementation Details

All samples from these five micro-expression datasets undergo the same data preprocessing procedure, with facial regions cropped to 224×224 pixels. In this study, we employ two different input modalities to comprehensively evaluate model performance: RGB apex frames and optical flow images. The RGB apex frames capture spatial texture features at the peak expression intensity, while the optical flow images, computed between onset and apex frames using the Recurrent All-Pairs Field Transforms (RAFT) algorithm [41], characterize temporal motion patterns of micro-expressions. Since the AU-guided augmentation strategy specifically operates on single-frame AU features, data augmentation is exclusively applied to RGB apex frame inputs.

All experiments are implemented using the PyTorch framework (Version: 2.3.1) and evaluated on three backbone networks: CNN, ResNet18, and PoolFormer-S12. The models are trained for 100 epochs with an initial learning rate of 0.0002 and weight decay of $5\times 10^{-4}$. To ensure stable optimization, the BRL loss is incorporated after the 50th epoch, and the best performance after this point is recorded. To systematically evaluate the effect of the proposed BRL module, we compare five different weighting coefficients λ=[0,0.3,0.5,0.7,0.9] in the total loss function $L_{\mathrm{total}}=\left(1-\lambda \right)L_{CE}+\lambda L_{BRL}$, where λ=0 serves as the baseline without BRL regularization. All hyperparameters are tuned to achieve optimal performance across different experimental settings.

### 4.3. Evaluation Metrics

To comprehensively evaluate model performance in cross-dataset micro-expression recognition, we employ accuracy (Acc) alongside two additional metrics that account for class imbalance: unweighted average recall (UAR) and unweighted F1-score (UF1). These metrics are defined as follows:

(7) $$\left\{\begin{array}{cc} \mathrm{Acc} & =\frac{\sum_{c=1}^{C}TP_{c}}{N}\\ \mathrm{UAR} & =\frac{1}{C}\sum_{c=1}^{C}\frac{TP_{c}}{N_{c}}\\ \mathrm{UF}1 & =\frac{1}{C}\sum_{c=1}^{C}\frac{2\times TP_{c}}{2\times TP_{c}+FP_{c}+FN_{c}} \end{array}\right.$$

where $TP_{c}$, $FP_{c}$, and $FN_{c}$ represent true positives, false positives, and false negatives for class *c*, respectively; $N_{c}$ denotes the total number of samples in class *c*; *N* is the total number of samples; and *C* is the number of classes. While Acc provides an overall performance measure, UAR and UF1 offer more balanced assessments by giving equal weight to each emotion category, thus mitigating the bias to majority classes that commonly exists in imbalanced micro-expression datasets.

### 4.4. Ablation Experiments

#### 4.4.1. Ablation of λ

An ablation study is conducted to analyze the influence of the weighting coefficient λ in the combined loss function $L_{\mathrm{total}}=\left(1-\lambda \right)L_{\mathrm{CE}}+\lambda L_{\mathrm{BRL}}$. The experiment uses apex frame inputs on the CNN backbone and is evaluated on the CASME II dataset. The parameter λ is tested with values of [0,0.3,0.5,0.7,0.9], where larger values indicate greater emphasis on the batch regularization loss component.

As shown in Table 4, the optimal performance is achieved at λ=0.5, where the model attains 56.59% Acc, 43.07% UAR, and 41.89% UF1. Compared to the baseline without BRL module (λ=0), this configuration yields improvements of 8.53% in Acc, 2.73% in UAR, and 2.70% in UF1. This balanced weighting allows the model to maintain strong classification capability while effectively utilizing the dataset-balancing regularization provided by the BRL module. When λ decreases to 0.3, the regularization effect is insufficient to adequately address feature distribution discrepancies across source domains, resulting in suboptimal performance. Conversely, when λ increases to 0.7 or 0.9, the excessive emphasis on dataset alignment compromises the model’s discriminative power for micro-expression classification, leading to significant performance degradation, with λ=0.9 performing even worse than the baseline case.

The performance trend across different λ values—rising to an optimum at λ=0.5 and then declining sharply at λ=0.7 and 0.9—reveals a critical trade-off in domain generalization. A low λ provides insufficient regularization to align features across domains, limiting generalization, while a high λ overly suppresses discriminative power for emotion classification, causing performance to fall below the baseline. These results confirm that an appropriate balance between classification learning and domain-invariant feature learning is essential for effective cross-dataset micro-expression recognition. The λ=0.5 configuration achieves this optimal trade-off, and its equal weighting (1:1) between the cross-entropy loss and the BRL loss is adopted in all subsequent experiments.

#### 4.4.2. Analysis with Apex Frame Inputs

This section presents the experimental results under the apex frame input setting, evaluating cross-dataset recognition performance across different datasets, backbone architectures, and the effect of the BRL module. Table 5 summarizes the comprehensive comparison, where bold values indicate the best performance for each dataset under each evaluation metric.

At the dataset level, CASME achieves the highest recognition Acc among all test configurations, reaching 69.88% with both CNN+BRL and ResNet+BRL. This superior performance can be attributed to the exclusion of CASME during training, which results in the largest composite training set among all test scenarios due to CASME’s relatively small sample size. In contrast, SMIC consistently exhibits the lowest recognition accuracy across all configurations, a trend that persists throughout the subsequent experimental results. This performance gap stems from two primary factors. First, SMIC has a relatively small training sample size, which limits the amount of representative data available for model optimization. Second, and more critically, SMIC does not provide explicit apex frame annotations. The use of detected apex frames—defined as the frame with the largest motion magnitude within the sequence—introduces potential temporal misalignment, particularly affecting optical flow features that rely on precise onset–apex timing. These limitations collectively hinder the model’s ability to learn robust and well-aligned features from this dataset.

Regarding backbone architectures, CNN demonstrates the most substantial improvements when integrated with the BRL module, with average performance increases of 6.95% in Acc, 6.62% in UAR, and 8.70% in UF1. Both CNN and ResNet significantly outperform PoolFormer across most evaluation metrics. Overall, the CNN architecture combined with the BRL module delivers the best performance across most datasets (except CASME), demonstrating its superior capability in handling cross-dataset micro-expression recognition tasks when using apex frames as input.

The incorporation of the BRL module effectively enhances model performance across all backbone networks. The most notable improvement is observed in the CNN architecture, where the addition of BRL boosts the average accuracy from 52.28% to 59.23%. This demonstrates the effectiveness of the proposed batch regularization learning in addressing feature distribution discrepancies across datasets, particularly for architectures with moderate complexity that are well-suited for the scale of available micro-expression data.

#### 4.4.3. Analysis with Optical Flow Inputs

This section evaluates the effectiveness of temporal motion features for cross-dataset micro-expression recognition, using optical flow sequences computed between onset and apex frames via the RAFT algorithm [41]. Table 6 presents the comprehensive results, with bold values indicating the best performance for each dataset under each evaluation metric.

Compared to the apex frame inputs analyzed in Section 4.4.2, optical flow inputs significantly outperform apex frame inputs (Table 5) across all evaluation metrics and backbone architectures. This performance advantage demonstrates that temporal motion patterns capture more robust and transferable characteristics for cross-dataset recognition compared to spatial appearance features from single frames. Among the architectures, the PoolFormer architecture exhibits the most substantial improvement with optical flow inputs, achieving performance increases of over 10% across respective metrics. The incorporation of the BRL module further enhances model performance with optical flow inputs across all backbone networks. The CNN architecture achieves the most consistent improvements, with performance gains of 3.76% in Acc, 4.26% in UAR, and 3.59% in UF1 after BRL integration. While the PoolFormer+BRL configuration delivers particularly competitive results on SMIC and SAMM datasets, CNN+BRL achieves superior performance in the remaining cases.

These findings collectively demonstrate that optical flow features provide more discriminative temporal representations than single apex frames for cross-dataset micro-expression recognition. The superior generalizability of optical flow stems from its inherent robustness to cross-domain variations. Unlike appearance features from single apex frames, which are sensitive to illumination and camera differences, optical flow encodes relative motion, thereby normalizing dataset-specific biases. Micro-expressions are transient motion events, and optical flow directly captures the dynamics of facial muscle activations over time, which are more consistent across data collection setups. Consequently, models learn transferable motion patterns rather than appearance artifacts, leading to stronger generalization to unseen domains. Moreover, the BRL module effectively enhances model performance with different input types and architectural designs, confirming its robustness and general applicability across varied experimental conditions.

#### 4.4.4. Ablation of Data Augmentation

This section evaluates the proposed AU-guided data augmentation strategy, which generates synthetic samples based on clustered AU intensity patterns for each micro-expression category. As the augmentation operates on single-frame AU characteristics, we validate its effectiveness using apex frame inputs across the three backbone architectures with BRL module integration. Table 7 presents the results with data augmentation, where bold values indicate the best performance for each dataset under each evaluation metric.

When comparing with the non-augmented results in Table 5, the integration of AU-guided data augmentation yields significant performance gains, with particularly notable improvements in UAR and UF1 metrics. This trend appears across all three backbone architectures. The CNN+BRL configuration with augmentation demonstrates remarkable gains on the MMEW dataset, achieving 6.40% higher UAR and 11.02% better UF1 compared to the non-augmented case. At the architecture level, ResNet+BRL+Augmentation shows the most substantial average improvements with 3.95% higher UAR and 5.28% better UF1, followed by PoolFormer and CNN. The pronounced enhancement in UAR and UF1, which are metrics specifically designed to evaluate performance on imbalanced datasets, confirms that the augmentation strategy effectively mitigates inter-dataset sample quantity disparity by generating representative samples that better capture characteristic AU patterns for each emotion category.

These results demonstrate that the AU-guided data augmentation effectively addresses dataset imbalance in cross-dataset micro-expression recognition. The method generates semantically consistent samples by leveraging statistically derived AU intensity centroids from real data distributions. This approach maintains computational efficiency through single-pass generation via the ULME-GAN network while specifically targeting class imbalance at the AU level. By preserving essential facial action characteristics of each emotion category, the augmentation diversifies training data while ensuring feature authenticity.

#### 4.4.5. Ablation Comparison and Visualization

To comprehensively evaluate the overall effectiveness of the proposed modules, we analyze the average cross-dataset recognition performance across all five datasets using the CNN backbone, which has demonstrated superior performance in previous experiments. Table 8 presents the ablation results of different module combinations, revealing that both the BRL module and data augmentation contribute significantly to performance improvement.

The results in Table 8 indicate a clear performance hierarchy among different configurations. The baseline model using apex frames without any proposed modules achieves 52.28% Acc, 40.13% UAR, and 36.46% UF1. Incorporating the BRL module brings substantial improvements, increasing these metrics to 59.23%, 46.75%, and 45.16% respectively. The addition of data augmentation further enhances performance, particularly on UAR and UF1, which rise to 49.10% and 47.83%. Notably, optical flow features outperform apex frames across all configurations, with the combination of optical flow inputs and BRL module achieving the best overall performance at 63.50% Acc, 53.63% UAR, and 53.07% UF1.

**Figure 7:**
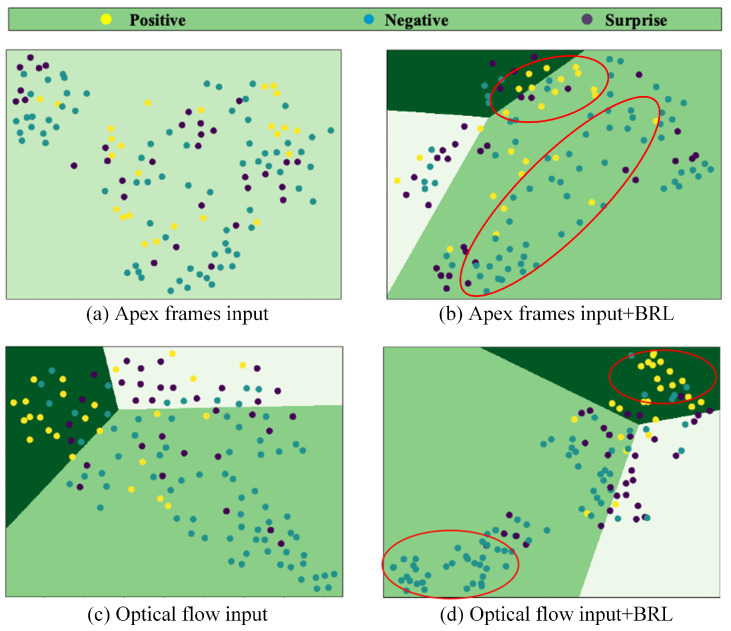
Visualization using logistic regression for features extracted by a CNN trained on four datasets and tested on CASME II. The red circles highlight the areas where samples are clustered. (**a**) Classification using apex frames; (**b**) Classification using apex frames with BRL Loss; (**c**) Classification using optical flow features; (**d**) Classification using optical flow features with BRL module.

We further investigate the impact of BRL through feature visualization. For each trained model, test samples are processed to extract features, which are then reduced to 2D space using Principal Component Analysis (PCA). A logistic regression classifier is trained on these reduced-dimension features to identify and plot decision boundaries. Figure 7 presents the visualization results under four different configurations.

The visualization reveals distinct patterns in feature learning. For apex frame inputs (Figure 7a), the feature distribution appears chaotic without BRL, making it difficult to establish clear decision boundaries. Although BRL integration (Figure 7b) brings moderate improvement in feature clustering, significant inter-class mixing persists, indicating the limited discriminative capacity of spatial features alone in cross-dataset scenarios. In contrast, optical flow inputs (Figure 7c,d) demonstrate better inherent separability, with the BRL module further enhancing class discrimination. Particularly in Figure 7d, the feature clusters become more compact and well-separated, as highlighted by the red circles, indicating that the combination of optical flow features and BRL regularization effectively learns discriminative and dataset-invariant representations. This advantage stems from the fundamental characteristic that optical flow features directly encode temporal dynamics of facial muscle movements, which captures the essential nature of micro-expressions more effectively than static texture features from apex frames. Furthermore, motion patterns exhibit greater robustness to inter-dataset appearance variations such as illumination and subject demographics.

These experimental results demonstrate that the proposed methods effectively address feature distribution inconsistency and data imbalance in cross-dataset micro-expression recognition, with optical flow features providing superior temporal representations and the BRL module enhancing feature discriminability across diverse dataset distributions.

### 4.5. Comparison with Other Methods

Due to the relatively limited research on cross-dataset micro-expression recognition and the inconsistent use of evaluation datasets across existing studies, we compare our method with available state-of-the-art approaches under the same tested dataset. Since most existing methods primarily report Acc and UAR with one decimal place precision, Table 9 presents our best results rounded to one decimal place for comparison. Bold values indicate the best performance for each metric across different datasets.

As shown in Table 9, our method achieves superior performance across most datasets and evaluation metrics. On the CASME II dataset, our approach attains 73.6% Acc and 68.9% UAR, surpassing the best existing methods by 7.4% in Acc compared to DR [34] and by 4.6% in UAR relative to ATNet [31]. Similarly, on the SAMM dataset, our method reaches 67.8% Acc and 53.2% UAR, representing an 11.9% improvement in Acc over RNMA [42] and a 7.4% gain in UAR compared to ATNet [31]. However, on the SMIC dataset, our method does not achieve the best performance, obtaining 51.2% accuracy and 46.6% UAR. This performance limitation can be attributed to the absence of precise apex frame annotations in SMIC, where optical flow features are extracted using the middle frame of the sequence rather than accurately identified apex frames, potentially introducing temporal misalignment that compromises feature quality.

**Table 9:** Comparison with state-of-the-art methods (Acc and UAR). Bold values indicate the best results.

Method	SMIC		CASME II		SAMM	
	**Acc**	**UAR**	**Acc**	**UAR**	**Acc**	**UAR**
LBP-TOP [17]	-	-	23.2	31.6	33.8	32.7
3DHOG [17]	-	-	37.3	18.7	35.3	26.9
HOOF [17]	-	-	26.5	34.6	44.4	34.9
DR [34]	**54.9**	**54.7**	66.2	49.6	-	-
D3DCNN [43]	-	-	44.7	-	36.9	-
TFMVN [44]	-	-	45.5	36.7	-	-
ELRCN [45]	-	-	38.4	32.2	48.5	38.2
RN [26]	-	-	57.8	33.7	54.4	44.0
RNMA [42]	-	-	58.4	34.1	55.9	42.7
RSTR [33]	45.1	-	56.2	-	-	-
ATNet [31]	-	52.3	-	64.3	-	45.8
Ours	51.2	46.6	**73.6**	**68.9**	**67.8**	**53.2**

These results demonstrate that the proposed BRL module and AU-guided data augmentation effectively address the core challenges in cross-dataset micro-expression recognition: feature distribution inconsistency and data imbalance. Notably, these improvements are achieved through targeted methodological innovations rather than increased architectural complexity. The performance superiority across datasets confirms that our approach learns discriminative and dataset-invariant feature representations, demonstrating both effectiveness and strong generalizability for micro-expression recognition.

## 5. Conclusions and Discussion

Cross-dataset micro-expression recognition presents a critical challenge for real-world applications, where models must maintain performance when encountering data from previously unseen sources. To address this challenge systematically, this study establishes a rigorous evaluation paradigm using five publicly available spontaneous micro-expression datasets under a leave-one-dataset-out protocol. This framework reveals two fundamental difficulties in cross-dataset scenarios: feature distribution inconsistency across source domains and inherent dataset imbalance. We propose targeted solutions for each challenge and demonstrate state-of-the-art performance across multiple evaluation metrics.

Through comprehensive experimental analysis and visualization, we identify that feature distribution shifts across datasets cause models to learn dataset-specific biases rather than generalizable micro-expression characteristics. Additionally, the natural imbalance in sample quantities among different datasets introduces training biases that further degrade cross-dataset generalization capability. These interrelated issues significantly impact model performance in practical cross-dataset applications. The core of the problem lies in the model’s inefficient information acquisition strategy. It tends to overfit to high-entropy but domain-specific signals, while underutilizing the essential, low-entropy stable emotional cues that are consistent across domains.

To address feature distribution inconsistency, we propose BRL as a plug-and-play learning strategy that enhances model generalization by explicitly balancing its attention across multiple source domains. This approach adaptively adjusts feature importance during training to encourage domain-invariant representation learning. The BRL module functions as an information flow regularizer, minimizing the entropy in attention distribution across domains and promoting a more equitable extraction of information from all available sources. The modular design enables integration into various backbone architectures and facilitates straightforward transfer to similar cross-domain recognition tasks. For the dataset imbalance problem, we introduce an AU-guided data augmentation strategy that generates semantically consistent samples based on clustered AU intensity patterns. Importantly, these solutions achieve significant performance improvements on three conventional backbone networks (CNN, ResNet, and PoolFormer), demonstrating that our methods effectively address the core challenges without relying on architectural complexity.

Despite the promising results, several limitations remain. Performance variations across different test datasets indicate that more universal micro-expression representations need to be developed. Future work will focus on advanced temporal feature learning techniques and domain generalization methods to create more robust cross-dataset recognition systems. The exploration of self-supervised learning paradigms and the integration of physiological prior knowledge also present promising directions for enhancing cross-dataset micro-expression recognition.

## Figures and Tables

**Table 2:** Number of augmented samples per category for each dataset.

Dataset	Positive	Negative	Surprise	Total
SMIC	23	23	24	70
CASME	51	50	50	151
CASME II	35	35	35	105
SAMM	38	37	38	113
MMEW	-	-	-	-

**Table 3:** Comparison of training sample sizes with and without data augmentation for cross-dataset evaluation.

Test Dataset	Composite Training Dataset	Total Training Samples (Without Data Augmentation)	Total Training Samples (With Data Augmentation)
SMIC	CASME, CASME II, SAMM, and MMEW	567	936
CASME	SMIC, CASME II, SAMM, and MMEW	648	
CASME II	SMIC, CASME, SAMM, and MMEW	602	
SAMM	SMIC, CASME, CASME II, and MMEW	610	
MMEW	SMIC, CASME, CASME II, and SAMM	497	

**Table 4:** Performance comparison with different λ values on CASME II dataset using apex frames and CNN backbone.

λ	Acc	UAR	UF1
0	48.06	40.34	39.19
0.3	53.49	42.82	41.53
0.5	56.59	43.07	41.89
0.7	52.71	41.37	40.03
0.9	44.19	38.61	37.05

**Table 5:** Classification results of apex frame inputs across datasets with different backbones and BRL configurations. Bold values indicate the best performance for each metric within each dataset.

Method	Metric	SMIC	CASME	CASME II	SAMM	MMEW	Average
CNN	Acc	43.29	68.67	56.59	47.11	45.73	52.28
	UAR	36.85	45.63	43.07	42.30	32.79	40.13
	UF1	32.90	46.30	41.89	39.63	21.57	36.46
CNN+BRL	Acc	**46.95**	69.88	**62.79**	**66.94**	**49.57**	**59.23**
	UAR	**44.61**	51.25	**56.92**	**44.21**	**36.78**	**46.75**
	UF1	**41.59**	52.28	**56.61**	**44.04**	**31.27**	**45.16**
ResNet	Acc	46.34	63.86	48.06	61.98	42.74	52.60
	UAR	43.06	44.28	33.92	36.52	37.19	38.99
	UF1	39.62	44.50	33.57	35.69	36.45	37.97
ResNet+BRL	Acc	46.95	**69.88**	58.91	60.33	47.44	56.70
	UAR	40.52	**53.37**	38.66	40.31	34.77	41.53
	UF1	37.08	**54.28**	34.56	38.19	25.23	37.87
PoolFormer	Acc	40.24	69.88	51.16	55.37	36.75	50.68
	UAR	34.77	45.30	38.09	31.45	31.80	36.28
	UF1	29.85	46.37	38.13	30.37	20.70	33.08
PoolFormer+BRL	Acc	37.20	68.67	55.81	65.29	40.17	53.43
	UAR	31.62	50.82	37.39	32.92	32.53	37.06
	UF1	26.28	53.11	33.21	26.33	32.63	34.31

**Table 6:** Classification results of optical flow inputs across datasets with different backbones and BRL configurations. Bold values indicate the best performance for each metric within each dataset.

Method	Metric	SMIC	CASME	CASME II	SAMM	MMEW	Average
	Acc	44.51	66.27	68.99	58.68	60.26	59.74
	UAR	42.64	49.37	62.66	41.06	51.10	49.37
CNN	UF1	41.08	50.16	62.79	41.98	51.39	49.48
	Acc	48.17	**73.49**	**73.64**	62.81	59.40	**63.50**
	UAR	47.22	**57.93**	**68.91**	40.62	**53.45**	**53.63**
CNN+BRL	UF1	45.79	**58.70**	**68.77**	39.70	52.39	**53.07**
	Acc	46.95	69.88	65.89	57.85	55.98	59.31
	UAR	44.40	52.41	52.40	42.37	44.53	47.22
ResNet	UF1	44.85	52.94	52.21	42.62	43.22	47.17
	Acc	48.78	72.29	68.99	64.46	60.26	62.96
	UAR	47.15	54.83	61.20	36.04	53.24	50.49
ResNet+BRL	UF1	47.47	54.25	61.58	33.80	**53.66**	50.15
	Acc	46.95	69.88	66.67	63.64	**63.25**	62.08
	UAR	43.66	45.30	58.66	38.14	49.38	47.03
PoolFormer	UF1	39.27	46.34	59.31	36.29	46.62	45.57
	Acc	**51.22**	68.67	68.99	**65.29**	61.97	63.23
	UAR	**46.60**	43.72	63.81	**45.18**	52.74	50.41
PoolFormer+BRL	UF1	**46.55**	44.46	63.93	**45.46**	53.01	50.68

**Table 7:** Classification results using apex frames as input with BRL module and AU-guided data augmentation on different backbones. Bold values indicate the best performance for each metric within each dataset.

Method	Metric	SMIC	CASME	CASME II	SAMM	MMEW	Average
CNN+BRL+Augmentation	Acc	45.73	69.88	59.69	**67.77**	**53.42**	**59.30**
	UAR	**45.02**	52.21	**51.88**	**53.21**	**43.18**	**49.10**
	UF1	**43.59**	53.57	**52.70**	**46.97**	**42.29**	**47.83**
ResNet+BRL+Augmentation	Acc	46.34	**69.88**	59.69	61.98	44.44	56.48
	UAR	42.50	**65.87**	48.40	36.44	34.21	45.48
	UF1	42.46	**57.46**	48.36	35.27	32.18	43.15
PoolFormer+BRL+Augmentation	Acc	**46.95**	67.47	**60.47**	63.64	46.58	57.02
	UAR	43.84	50.00	47.13	32.95	33.47	41.48
	UF1	36.23	51.89	43.39	28.28	33.78	38.72

**Table 8:** Cross-dataset micro-expression recognition results (averaged over five datasets) with CNN backbone under different configurations.

Input	BRL	Data Augmentation	Average Acc	Average UAR	Average UF1
Apex	-	-	52.28	40.13	36.46
Apex	✓	-	59.23	46.75	45.16
Apex	✓	✓	59.30	49.10	47.83
Optical Flow	-	-	59.74	49.37	49.48
Optical Flow	✓	-	63.50	53.63	53.07

## Data Availability

Data sharing is not applicable.
